# The Doctor and the Strike

**Published:** 1921-04-09

**Authors:** 


### The Doctor and the Strike.

The coal strike is an evidence of the widening scope of these annoying disturbances of industry. Ten years ago coal strikes were affairs which stirred the sympathies of the public or appealed to their sense of the dramatic. But to-day they have changed from the local scale to the national; we are all experts now, by mere newspaper reading, in industrial and economic questions which our fathers had not begun to face, and the shifting of the grounds of strikes from simple wages questions to matters of policy has made every citizen an arbitrator.

Strikes have thus for long been extending the area of interested persons. This latest one has a very direct interest to the medical profession. There is no reason either why the doctor's views should not be consulted, and, indeed, if the real question at issue is ever settled by the rational application of mind to the matter, it will be because the doctor's habitual considerations have had their share in the settlement. The coal strike is directly an after-war settlement which had sooner or later to be made. During the war the wages of many classes of workers (by no means all) had been raised in pursuit of the ever-rising cost of living. In no important case had they risen in exact proportion, and in some cases the lag was, of course, very great. Two facts have challenged the continuance of these increases, the stagnation of trade and the fall in the cost of living. Employers say they cannot get custom, especially foreign custom, if they are to pay such wages. In many occupations reductions have been accepted. They have been made on the thoroughly unscientific system of taking off what employers thought fit or employees would bear without revolt. To treat the matter in this way is to pretend that simplicity exists where it does not. To reduce wages may help industry for the moment, but suppose wages are reduced to the point at which the effectiveness of the worker will disappear, and liability to sickness and short time develop. The loss to industry then may be not only more serious but lasting.

During the past two years we have, as a nation, been better nourished than for many years. Observers have noted the growing development of the people since the war. No doubt this fact has enabled us to withstand not only the physical but the mental strain of these times. If the standard of life is to be suddenly lowered it may be that not only the bodily energy of the workers but their mental stability will give way also, and we may have a period of unrest and distrust which will work havoc in every way. The only man who can deal adequately with such questions is the doctor. He ought to be called in and his opinion should be taken on the question of an adequate standard of life. If after that it is found impossible to accept his recommendations, we shall at least know how to take steps to minimise the evil or to cope with the troubles that may follow.

